# Mindfulness, Compassion, and Self-Compassion Among Health Care Professionals: What's New? A Systematic Review

**DOI:** 10.3389/fpsyg.2020.01683

**Published:** 2020-07-31

**Authors:** Ciro Conversano, Rebecca Ciacchini, Graziella Orrù, Mariagrazia Di Giuseppe, Angelo Gemignani, Andrea Poli

**Affiliations:** Department of Surgical, Medical and Molecular Pathology and of Critical Care Medicine, University of Pisa, Pisa, Italy

**Keywords:** mindfulness, compassion, self-compassion, empathy, health care, health care professional

## Abstract

Health care professionals (HCPs) are a population at risk for high levels of burnout and compassion fatigue. The aim of the present systematic review was to give an overview on recent literature about mindfulness and compassion characteristics of HCPs, while exploring the effectiveness of techniques, involving the two aspects, such as MBSR or mindfulness intervention and compassion fatigue-related programs. A search of databases, including PubMed and PsycINFO, was conducted following the Preferred Reporting Items for Systematic Review and Meta-Analyses (PRISMA) guidelines and the methodological quality for this systematic review was appraised using AMSTAR-2 (A MeaSurement Tool to Assess systematic Reviews-2). The number of articles that met the inclusion criteria was 58 (4 RCTs, 24 studies with pre-post measurements, 12 cross-sectional studies, 11 cohort studies and 7 qualitative studies). MBSR intervention was effective at improving, and maintaining, mindfulness and self-compassion levels and to improve burnout, depression, anxiety, stress. The most frequently employed interventional strategies were mindfulness-related trainings that were effective at improving mindfulness and self-compassion, but not compassion fatigue, levels. Compassion-related interventions have been shown to improve self-compassion, mindfulness and interpersonal conflict levels. Mindfulness was effective at improving negative affect and compassion fatigue, while compassion satisfaction may be related to cultivation of positive affect. This systematic review summarized the evidence regarding mindfulness- and compassion-related qualities of HCPs as well as potential effects of MBSR, mindfulness-related and compassion-related interventions on professionals' psychological variables like mindfulness, self-compassion and quality of life. Combining structured mindfulness and compassion cultivation trainings may enhance the effects of interventions, limit the variability of intervention protocols and improve data comparability of future research.

## Introduction

Mindfulness has been defined as the awareness that arises by intentionally paying attention, in the present moment and in a non-judgmental way, to the flow of experience (Kabat-Zinn, 2003). Mindfulness is a theoretical instance that finds its implementation in a set of meditation and psycho educational exercises aimed at understanding certain fundamental themes such as stress, attachment and dependence on internal and external content and therefore the importance of subjective observation of thoughts, emotions, and physical sensations (Kabat-Zinn, 2009; Rosa et al., 2019). Mindfulness propensity is known as facilitators of well-being and adaptation for healthcare professionals, together with high adaptive defense mechanisms (Catalano et al., 2019; Di Giuseppe et al., 2019a, 2020a,c; Martino et al., 2020a). Moreover, mindfulness interventions proved to be very helpful in reducing the psychopathological symptomatology of chronic conditions (Bonadonna, 2003; Catalano et al., 2017; Poli et al., 2017, 2019; Conversano and Marchi, 2018; Marchini et al., 2018; Conversano, 2019; Di Giuseppe et al., 2019b, 2020b; Martino et al., 2019a,b, 2020b; Merlo, 2019; Conversano et al., 2020a; Lenzo et al., 2020; Poli et al., 2020).

The most widespread protocol used both in the clinical and in the normal context is represented by a group-based intervention named Mindfulness-Based Stress Reduction (MBSR). It is a protocol characterized by the teaching of formal and informal meditation practices, yoga exercises and the sharing of group experiences (Kabat-Zinn, 2009). The aim of MBSR is to improve mindfulness ability in participants, helping them to integrate their mind and body activity in a non-judgmental way. Starting from the MBSR program, in the following years, many protocols based on it have been developed for research purposes, integrating various aspects of psychological and behavioral sciences (Shonin et al., 2013).

A plethora of research show that mindfulness-based interventions (MBIs) determine significant benefits both in clinical and non-clinical samples. On the one hand, MBIs would seem to increase awareness levels, strategies to cope with stressful situations and emotion management; on the other hand, they seem to reduce levels of perceived stress, anxiety, and depressive symptoms (Brown and Ryan, 2003; Cahn and Polich, 2006; Chiesa and Serretti, 2011). Generally, MBIs have shown a significant correlation with positive improvements in the following areas: attention, cognition, behavior and physiological processes which probably influence the functioning of the individual and their quality of life (Veltri et al., 2012; Jha et al., 2019; Marchi et al., 2019; Marazziti et al., 2020).

Tang et al. (2018), in their review, have emphasized how mindfulness acts mainly on three systems, such as attentional control, emotional regulation and self-awareness. The authors also highlighted that mindfulness practice can produce changes in both density and volume in gray matter, finding the following areas involved in mindfulness meditation: various prefrontal regions, anterior cingulate cortex, medial prefrontal cortex, striatum, amygdala, insula, posterior cingulate cortex and precuneus. Furthermore, the central effect that mindfulness practice plays on stress axes, together with plastic brain aspects elicited by practice and positive regulation of the immune system activity, may be conceptualized as the outcome of brain modification through mindfulness. Furthermore, a recent review highlights how meditation can elicit a meta-functioning of awareness in those who practice, allowing an increased mind/body regulation system (Giannandrea et al., 2019).

In particular, mindfulness training seems to increase three qualities of attention: (1) Attentional stability, meant to focus sustained attention without wandering. It is estimated that the human mind gets distracted for about half of the waking hours; mindfulness, both state and trait, is associated with a reduction in mental digressions, bringing the person back to focus on the present (Killingsworth and Gilbert, 2010; Smallwood and Schooler, 2015); (2) voluntary control of attention or selective attention, the ability to direct attention to a specific topic rather than another in an appropriate and voluntary manner (Wadlinger and Isaacowitz, 2011); (3) efficiency of attention, or an economic use of cognitive resources, involving less expenditure of cognitive activities for an attention performance on a specific task. Since mindfulness is based on the ability to control and orientate attention, it was also associated with an improvement in efficiency (Cahn and Polich, 2009). Regarding neural networks (Menon and Uddin, 2010; Menon, 2011; Piccinni et al., 2012), several studies are investigating the effects of mindfulness on the default mode network (DMN), the neural processes underlying spontaneous thoughts and wandering mind. In psychopathology, the DMN is often found to be hyperactivated and hyperconnected (Whitfield-Gabrieli and Ford, 2012). Research investigating expert mindfulness meditators engaging in simple breathing practice showed that activity in brain regions associated with the DMN was present during mind wandering, and in salience network (SN) areas during awareness of mind wandering, while regions of the executive network were active during shifting and sustained attention (Hasenkamp et al., 2012; Scheibner et al., 2017; Orrù et al., 2020a,b), and the effects were modulated by lifetime meditation experience (Brewer et al., 2011; Hasenkamp and Barsalou, 2012). Furthermore, research has shown that mindfulness training can lead to changes in the SN that regulates the switching between DMN and central executive network. Following mindfulness meditation, but not relaxation training, central regions of the SN, the left anterior cingulate cortex (ACC) and insula, showed improved cerebral blood flow (Tang et al., 2015). Similarly, loving-kindness meditators showed a deactivation of main nodes of the DMN (Brewer et al., 2011) and, after few weeks of compassion training, novice meditators showed significant reductions of mind wandering (Jazaieri et al., 2014). Taken together, these results suggest that though the focus of compassion meditation does not involve regulating attentional processes on a specific object, compassion training does have an impact on attentional processes involved in DMN.

Mental training following a mindfulness-based program seems to increase cognitive abilities both as cognitive capacity and as cognitive flexibility. The first includes working memory and fluid intelligence, the second supports the adaptation of an individual using new answers and strategies. Mindfulness training is associated with an increased working memory capacity, cognitive flexibility and benefits on fluid intelligence (Roeser et al., 2013; Ruocco and Direkoglu, 2013; Jha et al., 2019). It also promotes creative thinking and problem-solving skills (Colzato et al., 2012; Ostafin and Kassman, 2012; Raffone and Srinivasan, 2017).

Moreover, through mindfulness programs, participants may notice a different emotional regulation and functioning style; studies on the subject shows that mindfulness influences emotions both in terms of psychological reaction to emotion and tone or emotional value (positive or negative emotions). The increase in emotional regulation, or the set of processes and behavioral and cognitive strategies through which individuals influence their own emotional states following a mindfulness treatment, is related to an enhancement cognitive control mechanisms that refer to areas of the cortex prefrontal, such as the dorsolateral prefrontal cortex and prefrontal cortex ventromedial, and the anterior cingulate cortex, which act on the areas of the limbic system, used to process affective stimuli, such as the amygdala and the hippocampus (Chambers et al., 2009; Dell'Osso et al., 2012; Hölzel et al., 2013).

Furthermore, researchers found mindfulness training associated with a lower reactivity to stressful situations and negative emotional stimuli as it would increase the individual's ability to judge the situation more objectively and more emotionally of a positive value compared to those of a negative type (Farb et al., 2007; Hülsheger et al., 2013). Mindfulness exercises are related to a lower activation of the hypothalamic-pituitary-adrenal axis and cortisol secretion, influencing the stress response and its regulation, leading to a greater brain neuroplasticity and slowing the brain aging process (Brewer et al., 2011; Hölzel et al., 2013; McEwen and Morrison, 2013; Creswell and Lindsay, 2014; Fox et al., 2014; Luders et al., 2015).

More recently, some authors observed the importance of the ethical framework around mindfulness experiences. In particular, the authors postulate a theory which highlights the interrelation between the ethical beliefs of the individual and his relationship to the environment; cultivating internal and external awareness seems to arouse a process of ethics embodied in moral cognition. In fact, the changes reported by literature in MBI participants involve a meta-awareness and a different perspective of the self, probably influencing the processing of morally-relevant situations and stimuli thus encouraging moral action (Sevinc and Lazar, 2019). However, HCPs often suffer from psychological distress and therefore experience a varied spectrum of symptoms related to this condition. Specifically, a situation of psychological stress is able to elicit sleep disturbances (Palagini et al., 2016), cognitive problems, post-traumatic stress disorder (Marazziti et al., 2008; Mula et al., 2008) and also a burnout condition (Carmassi et al., 2016, 2017b, 2018; Moss, 2017; Conversano et al., 2020b).

Three types of meditation are typically included among “mindfulness meditations” in the West, namely focused attention, open monitoring (both attentional practices), and loving-kindness and compassion (both constructive practices) (Salzberg, 2011; Germer and Siegel, 2014). Focused attention entails bringing your attention back, again and again, to the breath or another focal object; open monitoring (or choiceless awareness) involves noticing what is most salient and alive in your field of awareness, moment-to-moment; loving-kindness meditation entails intentionally cultivating happiness while compassion meditation is about cultivating goodwill in the face of suffering (Germer and Siegel, 2014). While attentional meditation practices (focused attention and open monitoring) require focusing on a focal object or on a component part of the self, like perception, emotion, cognition or intention, loving-kindness, and compassion meditation require the self as the object of practice (Olendzki, 2013; Germer and Siegel, 2014; Dahl et al., 2015).

In particular, compassion is defined by Paul Gilbert, and the Buddhist monk Choden, as a sensitivity to suffering in self and others with a commitment to try to alleviate and prevent it (Gilbert, 2018), while self-compassion is defined by Kristin Neff as “being open to and moved by one's own suffering, experiencing feelings of caring and kindness toward oneself, taking an understanding, non-judgmental attitude toward one's inadequacies and failures, and recognizing that one's experience is part of the common human experience” (Neff, 2003). Recent brain imaging research supports the notion of evaluating compassion as more emotionally engaging than mindfulness. Compassion practice was found to activate regions of the positive affect system, such as the medial orbitofrontal cortex, nucleus accumbens and the ventral striatum (Klimecki et al., 2013, 2014; Engen and Singer, 2015). Specifically, Engen and Singer (2015) compared compassion meditation in response to cognitive reappraisal and showed that, in response to empathic distress, compassion meditation activated brain systems associated to positive emotion, while cognitive reappraisal recruited cognitive control regions and reduced activation of the negative affect system regions. Research also pointed out that mindfulness practitioners show reduced activation and structural changes of the amygdala (Hölzel et al., 2013; Taren et al., 2013, 2015). Overall, these results suggest that beneficial effects of mindfulness and compassion practices may act through different mechanisms: mindfulness reduces negative affect system's activity while compassion increases activity of positive emotion brain systems.

Interestingly, it has been shown that transcutaneous vagus nerve stimulation is able to modulate DMN in major depressive disorder (Fang et al., 2016) and that increased vagal activity was associated with higher compassion levels (Stellar et al., 2015). It has been shown that adopting a true compassionate disposition when viewing pictures of people suffering activated the mesolimbic dopamine pathway (ventral tegmental area and the ventral striatum) implicated in reward and bond formation (Kim et al., 2009). More recently, research showed that the septal nuclei, another area that is relevant for reward and prosocial motivation, was the unique region that was typically activated across empathy for pain, anxiety and happiness. Septal activation in course of these empathic experiences was predictive of helping (Morelli et al., 2014). Remarkably, it was recently shown that optogenetic activation of gut-innervating vagal sensory neurons, namely the dorsolateral parabrachial-nigral projections, mimicked the rewarding effects of right vagus excitation and identifies dorsolateral parabrachial region as the mandatory retransmission region linking the right vagal sensory ganglion to dopamine cells in substantia nigra (Han et al., 2018).

Interestingly, studies investigating compassion are consistent with those emerging from research on the neurobiology of the parental brain. It has long been observed in animal models that both the septal nuclei and the mesolimbic dopamine pathway are implicated in motivating proactive offspring nurture (Champagne et al., 2001). Specifically, ventral tegmental area dopaminergic neurons projecting to the nucleus accumbens motivate caregiving (Numan and Stolzenberg, 2009). Recent neuroimaging evidence suggests that this system may support human parental motivation to nurture their toddlers (Mascaro et al., 2013; Rilling, 2013), increasing the probability that this system underpins the motivational quality of compassion (Preston and Hofelich, 2012) and that compassion coopts the systems that evolved for maternal attachment (Preston and de Waal, 2001).

While it is very important that health care professionals (HCPs) continue to work with empathy and compassion, since compassionate motivation may impinge on the same circuits of maternal attachment, there may be a cost to this work that is also closely related to past maternal experience of HCPs. The concept of compassion fatigue first emerged with the work of Charles Figley, who defined it as “the formal caregiver's reduced capacity or interest in being empathic or ‘bearing the suffering of clients’ and is ‘the natural consequent behaviors and emotions resulting from knowing about a traumatizing event experienced or suffered by a person’” (Figley, 1995, 2002; Di Giuseppe et al., 2018). Compassion fatigue can be defined as secondary traumatic stress resulting from knowing about a traumatizing event experienced by a significant traumatized or suffering person (Figley, 1995, 2002). Prevalence of compassion fatigue has been reported to range as 7.3 to 40%, while the prevalence of secondary traumatic stress has been reported to range from 0 to 38.5% in intensive care units (Mol et al., 2015). Compassion fatigue often is assumed to accompany another version of fatigue known as burnout. Specifically, hard workloads, lack of recognition for achievements, and social disconnection from the team can result in burnout, suggesting the need to reduce the hours supporting the patient for formal (Amankwaa, 2017; Mattioli et al., 2018; Castellanos et al., 2019; Allday et al., 2020) as well informal (Peetoom et al., 2016; Lethin et al., 2017; Hampton and Newcomb, 2018; Wood et al., 2018) caregivers. However, compassion for another may originate from compassion for self. Gustin and Wagner (2013) found that developing a compassionate self and the aptitude to be sensitive, non-judgmental and regardful toward oneself promotes a compassionate approach toward others. Thus, developing self-compassion may be an important hindering factor of compassion fatigue and promotion of compassionate care (Neff, 2009) and mindfulness and self-compassion may be appropriate target variables to alleviate work-related stress and promote compassionate caregiving in HCPs.

The aim of the present systematic review was to give an overview on recent literature about mindfulness, compassion and self-compassion characteristics and to investigate the effectiveness of techniques involving MBSR or mindfulness-related interventions and/or compassion- or self-compassion-related programs, to identify the interventions with the best level of evidence available to prevent, or mitigate, HCPs' burnout and to improve their levels of mindfulness and/or compassion- or self-compassion, emotional regulation, quality of life and well-being.

## Methods

This review was conducted according to the Preferred Reporting Items for Systematic Review and Meta-Analyses (PRISMA) (Liberati et al., 2009; Moher et al., 2009).

### Search Strategy

Searches were conducted using PubMed and PsycInfo databases. Searches were conducted from January 2004 to May 2020 and were limited to English and Italian language papers. PubMed and PsycInfo databases were searched by three independent reviewers (CC, RC, and AP) using a combination of the keywords “Health Personnel,” “Mindfulness,” “Self-Compassion,” “MBSR,” using the Boolean operators AND/OR.

### Study Selection

**Figure 1:**
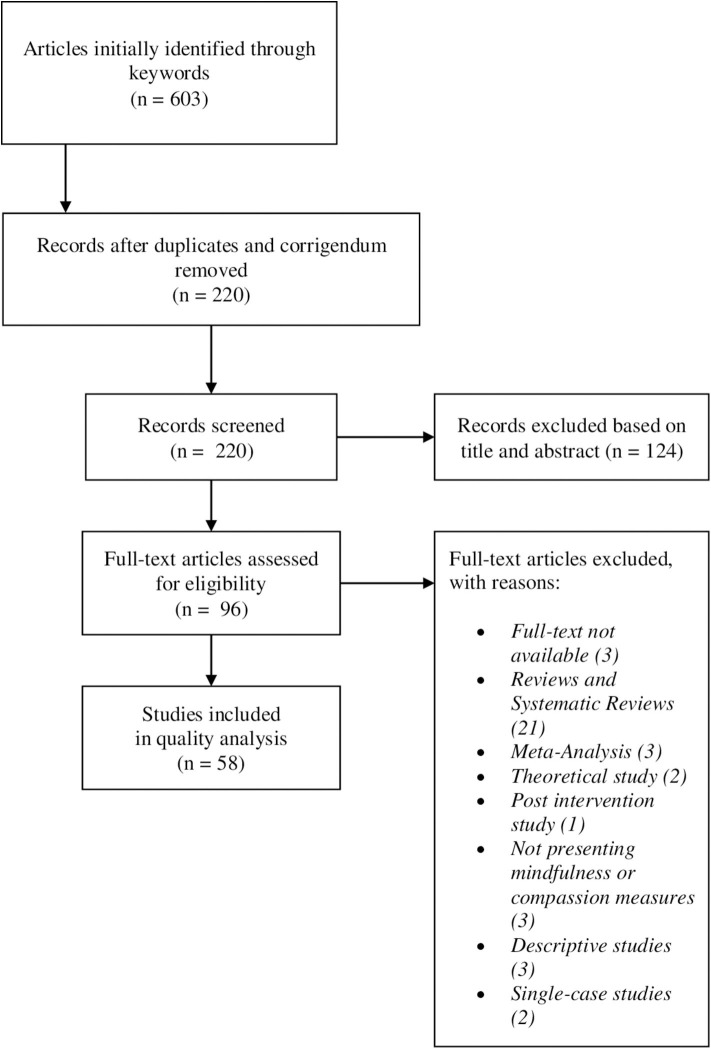
PRISMA Flow chart for literature search and screening results.

The selection of studies is shown in Figure 1, using the PRISMA flow diagram (Liberati et al., 2009; Moher et al., 2009). The search identified 603 articles, which were screened for eligibility. We removed duplicates and corrigenda (383) and then selected the articles which presented at least one or both mindfulness and compassion component and a healthcare unit/personnel/professional in title or abstract and removed those considered ineligible. Ninety-four full-text articles were retrieved and read in full by three reviewers (CC, RC, and AP) and divided by type and study design and, finally, we excluded descriptive, single case and post intervention studies and reviews. Qualitative studies are included. Studies that did not report measures of mindfulness and self-compassion were excluded. Overall, while the authors were independently reading the articles, 38 articles were excluded from the results due to (a) not in accordance with the main topic (b) study design not included (b) full text not available. A total of 58 studies were included in the review (Table 1).

**Table 1:** Summary of the revised studies.

	**Authors**	**Title**	**Year**
1	Allie et al.	Bereavement overload and its effects on, and related coping mechanisms of health care providers and ward administrators at National District Hospital in Bloemfontein, Free State	2018
2	Al-Majid et al.	Assessing the degree of compassion satisfaction and compassion fatigue among critical care, oncology, and charge nurses	2018
3	Amutio et al.	Acceptability and effectiveness of a long-term educational intervention to reduce physicians' stress-related conditions	2015
4	Brady et al.	The impact of mindfulness meditation in promoting a culture of safety on an acute psychiatric unit	2012
5	Brooker et al.	Evaluation of an Occupational Mindfulness program for staff employed in the disability sector in Australia	2013
6	Brown et al.	Compassion fatigue and mindfulness: comparing mental health professionals and MSW student interns	2017
7	Cohen-Katz et al.	The effects of mindfulness-based stress reduction on nurse stress and burnout: a quantitative and qualitative study	2004
8	Copeland and Henry	The relationship between workplace violence, perceptions of safety, and Professional Quality of Life among emergency department staff members in a Level 1 Trauma Centre	2018
9	Dev et al.	Does self-compassion mitigate the relationship between burnout and barriers to compassion? A cross-sectional quantitative study of 799 nurses	2018
10	dos Santos et al.	Positive effects of a stress reduction program based on mindfulness meditation in Brazilian nursing professionals: qualitative and quantitative evaluation	2016
11	Duarte and Pinto-Gouveia	Mindfulness, self-compassion and psychological inflexibility mediate the Brady effects of a mindfulness-based intervention in a sample of oncology nurses	2017
12	Duggan and Julliard	Implementation of a mindfulness moment initiative for healthcare professionals: perceptions of facilitators	2018
13	Eliassen et al.	The effect of training in mindfulness and affect consciousness on the therapeutic environment for patients with psychoses: an explorative intervention study	2016
14	Erkorkmaz et al.	The relationship between burnout, self-esteem and professional life quality of nurses	2018
15	Farina et al.	Introducing mindfulness practices for self-care: outcomes of a brief education session	2018
16	Fernando et al.	Increasing compassion in medical decision-making: can a brief mindfulness intervention help?	2017
17	Gracia-Gracia and Oliván-Blázquez	Burnout and mindfulness self-compassion in nurses of intensive care units: cross-sectional study	2017
18	Horner et al.	A pilot study to evaluate mindfulness as a strategy to improve inpatient nurse and patient experiences	2014
19	Hunter et al.	Being there and reconnecting: midwives' perceptions of the impact of Mindfulness training on their practice	2018
20	Ireland et al.	A randomized controlled trial of mindfulness to reduce stress and burnout among intern practitioners	2017
21	Kemper	Brief online mindfulness training: immediate impact	2017
22	Kemper and Rao	Brief online focused attention meditation training: immediate impact	2017
23	Kemper et al.	What is the impact of online training in mind-body skills?	2015
24	Kemper and Khirallah	Acute effects of online mind-body skills training on resilience, mindfulness, and empathy	2015
25	Klatt et al.	A pragmatic introduction of mindfulness in a continuing education setting: exploring personal experience, bridging to professional practice	2017
26	Lamothe et al.	Developing professional caregivers' empathy and emotional competencies through mindfulness-based stress reduction (MBSR): results of two proof-of-concept studies	2018
27	Luthar et al.	Fostering resilience among mothers under stress: “Authentic Connections Groups” for medical professionals	2017
28	Lyddy et al.	Transfer of mindfulness training to the work setting: a qualitative study in a health care system	2016
29	McPherson et al.	Distress in working on dementia wards—a threat to compassionate care: a grounded theory study	2016
30	Montero-Marin et al.	Burnout subtypes and absence of self-compassion in primary healthcare professionals: a cross-sectional study	2016
31	Morrison Wylde et al.	Mindfulness for novice pediatric nurses: smartphone application vs. traditional intervention	2017
32	Ofei-Dodoo et al.	Impact of a mindfulness-based, workplace group yoga intervention on burnout, self-care, and compassion in health care professionals: a pilot study	2020
33	Olson et al.	What factors promote resilience and protect against burnout in first-year pediatric and medicine-pediatric residents?	2015
34	O'Mahony et al.	A Multimodal Mindfulness training to address mental health symptoms in providers who care for and interact with children in relation to end-of-life care	2016
35	Orellana-Rios et al.	Mindfulness and compassion-oriented practices at work reduce distress and enhance self-care of palliative care teams: a mixed-method evaluation of an “on the job” program	2017
36	Pakenham et al.	Effects of Acceptance and Commitment Therapy (ACT) training on clinical psychology trainee stress, therapist skills and attributes, and ACT processes	2015
37	Pfaff et al.	Reducing the “cost of caring” in cancer care: evaluation of a pilot interprofessional compassion fatigue resiliency programme	2017
38	Pflugeisen et al.	Brief video-module administered mindfulness program for physicians: a pilot study	2016
39	Platt et al.	Fostering resilience with GPs: a workshop approach	2015
40	Powell et al.	Work-Life BALANCE: How Some Case Managers Do It!	2018
41	Raab et al.	Mindfulness-based stress reduction and self-compassion among mental healthcare professionals: a pilot study	2015
42	Riley et al.	Improving physical and mental health in frontline mental health care providers: yoga-based stress management vs. cognitive behavioral stress management	2017
43	Ripp et al.	Well-being in graduate medical education: a call for action	2017
44	Roney and Acri	The cost of caring: an exploration of compassion fatigue, compassion satisfaction, and job satisfaction in pediatric nurses	2018
45	Sanso et al.	Evaluation of a mindfulness intervention in palliative care teams [Spanish]	2018
46	Scarlet et al.	The effects of Compassion Cultivation Training (CCT) on health-care workers	2017
47	Shapiro et al.	Mindfulness-based stress reduction for health care professionals: results from a randomized trial	2005
48	Silver et al.	Mindfulness among genetic counselors is associated with increased empathy and work engagement and decreased burnout and compassion fatigue	2018
49	Steinberg et al.	Feasibility of a mindfulness-based intervention for surgical intensive care unit personnel	2016
50	Suyi et al.	Effectiveness of mindfulness intervention in reducing stress and burnout for mental health professionals in Singapore	2017
51	Taylor et al.	A mindfulness intervention for residents: relevance for pediatricians	2016
52	Valley and Stallones	A thematic analysis of health care workers' adoption of mindfulness practices	2018
53	Valley and Stallones	Effect of mindfulness-based stress reduction training on health care worker safety: a randomized waitlist controlled trial	2017
54	Verweij et al.	Mindfulness-based stress reduction for GPs: results of a controlled mixed methods pilot study in Dutch primary care	2016
55	Wacker and Dziobek	Preventing empathic distress and social stressors at work through non-violent communication training: a field study with health professionals	2018
56	Wahl et al.	Implementing a Peer Support Network to Promote Compassion Without Fatigue	2018
57	Wen et al.	Encouraging mindfulness in medical house staff via smartphone app: a pilot study	2017
58	Wijdenes et al.	Assessing compassion fatigue risk among nurses in a large urban trauma center	2019

### Eligibility Criteria

All studies included in our review examined the effect of MBSR, MBIs (interventions including predominantly mindfulness elements; Spinelli et al., 2019); and compassion-based interventions (CBIs, interventions including predominantly compassion elements; Kirby, 2017); on mindfulness and self-compassion qualities of HCPs. Interventions using yoga, or other meditative practices, and MBIs that used the key therapeutic approaches, such as body scan meditations or acceptance or non-judgmental strategies, were included.

### Data Extraction

At an earlier stage, titles and abstracts were screened for eligibility by one reviewer (CC) to characterize abstracts as highly relevant, potentially relevant, or not relevant. Full texts were then accessed for all abstracts characterized as highly or potentially relevant and read in full by three reviewers (CC, RC, and AP) to determine whether they were eligible. Studies that were considered ambiguous, with respect to the inclusion criteria, were discussed, and consensus was reached for all articles included.

The following data was extracted from each eligible study report by RC and verified by CC, GO, AG, or AP: first author name, title, publication date, aim and design, key sample characteristics (including age, gender, and healthcare profession), type of intervention, comparison, main outcome measures, and results.

### Quality Assessment

The appraisal of the methodological quality for this systematic review was based on AMSTAR-2 (A MeaSurement Tool to Assess systematic Reviews-2) (Shea et al., 2017) approach. It enables a more detailed assessment of systematic reviews that include randomized or non-randomized studies of healthcare interventions, or both. Two independent raters assessed each study (CC and RC), and seven discrepancies were found regarding AMSTAR-2 checklist coding. Then, a discussion took place to resolve divergences, consulting a third rater (AP), if necessary, until consensus was reached.

### Analysis

After examination of included articles, meta-analysis was not considered suitable as there were insufficient studies with the required level of homogeneity regarding MBIs, CBIs, outcome measures, and the timing of these measures. Therefore, the findings are summarized using a narrative, but systematic, approach.

## Results

The included experimental studies showed several different types of design such as: randomized controlled trials (4), studies with pre-post measurements (24), cross-sectional studies (12), cohort studies (11), and qualitative studies (7).

Among the included articles, some of the experimental studies investigated the effectiveness of an intervention based on MBSR (Kabat-Zinn, 2009) or compassion-related interventions (Neff and Germer, 2013) in relation to psychological variables and factors related to quality of life in HCPs. Some studies compared MBSR, mindfulness-related interventions, compassion-related interventions with untreated control groups, while other studies cross-sectionally probed mindfulness or compassion variables in the investigated sample, trying to find correlations with other professional variables such as burnout and empathy that did not undergo any kind of intervention. We highlight that some studies have concerned online interventions both in terms of their creation and impact on health outcomes.

### Qualitative Studies

With regard to mindfulness interventions, Ripp et al. (2017) highlight its importance in the fight against burnout in graduate medical trainees together with “resilience training, stress and self-care management workshops, communication skills training, narrative medicine, reflection opportunities and peer support grounded in group discussions.”

Hunter et al. (2018) conducted a qualitative study through an interpretative phenomenological analysis of semi-structured interviews. The measures were operated on midwives after a MBI, either at or outside workplace. Nine midwives selected to take part in the study, and the findings were divided in four superordinate themes: being challenged and committing, containing the self, reconnecting and moving forward with confidence. Results showed that, despite an initial and normal skepticism about mindfulness practice, those that committed to the concept of living in the present moment benefitted from an increased self-awareness of self which presumably leads to both an increase of positive workplace relationships and individualized and relation patient care.

The research trio of McPherson et al. (2016) conducted a study to explore the experiences of managing work pressures of staff caring for older adults with dementia through compassion and mindfulness skills. The interviewer used a constructivist grounded theory approach which is guided by participants themselves, finding two main types of work-related pressure (structural or interpersonal) and two different types of responses (helpful and unhelpful). In the helpful strategies, taking a moment, talking, reflecting, and processing were included, but did not reach a sufficient level to be considered practice. The authors also found some barriers to the full experience of the self-compassionate and mindful state, which leads to their suggestion of increasing structured mindfulness interventions in HCPs.

Platt et al. (2015) wrote a qualitative paper about a workshop which was planned for general practitioners, comprehending stress management sessions, art or puppetry and mindfulness sessions. This workshop's aim was to help the participants develop a better self-awareness when facing stress situations and identify different ways to manage the stress response. The 25 participants were interviewed during the workshop and 2 months after it (by an online survey), for feedback. Results showed that this kind of intervention was well-received, with immediate to medium-term impact on the individual.

Lyddy et al. (2016) explored how health professionals, which had previously followed a mindfulness-based course, and use and perceive mindfulness practice during their work. The measurement was carried out through a semi-structured guide with open-ended questions, also including questions about role and recent prototypical job experiences. Their results highlight that participants varied in the subsequent adoption of mindfulness exercises, often struggling with the formal meditation practice routine and using the informal practice models taught during the course. The authors suggested that mindfulness training in hospitals should include practice in the actual workplace to increase realism and integration of practice on a daily basis and practice in identifying the value of mindfulness at work through guided experimentations.

In their interesting work, Valley and Stallones (2018) also aimed at confirming the role of mindfulness interventions in the development of self-awareness, using the Health Belief Model (HBM) to help explain and predict the adoption of a variety of preventive and treatment health behaviors. In their research of what is needed to improve mindfulness interventions in health care workers, they found that adapting the course timing and materials to meet the HCPs' schedule and a didactic material which explains the evidence of those interventions should be provided to the participant in order to increase the participation and adherence to the courses.

Looking at the issue from the opposite point of view, Powell et al. (2018) have raised the question of what makes some health professionals more protected from the risk of burnout than others. In her work, she asked through a convenience survey, administered to some multidisciplinary staff members about their activities outside work which “provides a balance to the daily triumphs and challenges of their work–life in case management” and if the activity “enhance their life at work” and how. Although only 32 of those staff members replied to the survey, we can highlight that the findings included meditation, mindfulness, exercise, nature and animals, family and friends as activities indicated by the participants which could lead to some sort of balance during work.

### Randomized Controlled Trials

Shapiro et al. (2005) conducted a RCT on the effectiveness of MBSR training in a population of HCPs, directly involved in clinical work, in particular measuring the stress and distress response and job burnout. For the MBSR participants, lower stress level and higher self-compassion was reported, with a reduction of job burnout. Unfortunately, these results didn't differ significantly from the control group, contrary to their qualitative data.

Amutio et al. (2015) tested both acceptability and effectiveness of an MBSR with a maintenance phase (8 week traditional intervention plus 10 month of maintenance) with the aim to alleviate work-stress related symptoms such as burnout and blood pressure levels. Their findings showed decreased burnout (especially emotional exhaustion subscale), blood pressure and heart rate, which were still decreasing after 10 months from the MBSR programme. Similarly, Ireland et al. (2017) tested the effectiveness of a mixed mindfulness intervention in reducing stress and burnout among doctors assigned to an emergency department rotation programme. Also, their findings suggested a decreased stress and burnout of the participants, to a greater extend that an assigned extra break hour for 10 weeks.

Finally, Valley and Stallones (2017) RCT study examined the impact of mindfulness training on occupational safety of hospital HCPs, through an MBSR intervention, measuring safety outcomes (compliance and participation). Their results indicated that mindfulness was able to stably decrease workplace cognitive failures while increasing the following of safety rules, collaboration, and safety compliance behaviors. The authors highlight the importance of these findings which could impact the degree of occupational injuries.

### Cross-Sectional Studies

#### Understanding the Link Between Compassion Fatigue and Compassion Satisfaction, Bereavement Overload, Burnout, and Violence

Wijdenes et al. (2019) conducted a research with the aim of exploring the prevalence and severity of compassion fatigue risk among nurses and to ascertain the differences in demographic characteristics among the participants correlating with the abovementioned risk. Their measures included the Professional Quality of Life version 5 (ProQOL-5) (Stamm, 2009) and their findings were that nearly half of the sample was at risk for moderate to high compassion fatigue. Moreover, nurses' unit was significantly associated with compassion satisfaction while time and experience in service were not protective factors for compassion fatigue. Lastly, secondary traumatic stress, burnout and compassion satisfaction were significantly correlated.

Al-Majid et al. (2018) assessed the degree of compassion satisfaction and compassion fatigue in both care and charge nurses of care units. The measurement was conducted through the ProQOL-5, leading to an average overall result concerning compassion variables with significantly lower compassion satisfaction in less experienced nurses (<10 years of work), while other scales such as secondary traumatic stress showed high levels among charge nurses.

Likewise, Roney and Acri (2018) investigated pediatric nurses' levels of compassion satisfaction, compassion fatigue, and job satisfaction while considering any correlations among the constructs, using the ProQOL-5. Their results found participants with higher than the norm levels of compassion satisfaction and slightly lower than the norm levels of secondary trauma and burnout, highlighting an interesting significant relationship between female gender and the compassion satisfaction subscale.

Allie et al. (2018) focused their cross-sectional analytic study on HCPs' bereavement overload, defined as a situation where an individual must deal with loss or death in a continuous and close way with the unfortunate result of an abnormal adjustment process. Their study investigated through an interviewer-administered questionnaire various quantitative variable such as bereavement overload, compassion fatigue and coping mechanisms. Findings showed that half of the participants reported a suffering from bereavement overload, of which three quarters reported compassion fatigue (especially doctors and final-year medical students). Interestingly, also half of the health providers which didn't show bereavement overload, suffered from compassion fatigue. The authors have deduced that while compassion fatigue may be an effect of bereavement overload it could lead to dysfunctional coping mechanisms such as emotional detachment.

Copeland and Henry (2018) designed a cross-sectional study to survey emergency department staff members with the purpose of examining the relationship between exposure to workplace violence (physical and psychological) and compassion fatigue or satisfaction through the Professional Quality of Life model. The findings of this study showed that all three of the ProQOL dimensions were significantly associated with exposure to violence, particularly with patient threats, name calling, sexual innuendo and threats of lawsuit. Furthermore, as the study described above (Al-Majid et al., 2018), also from their findings the most experienced nurses in the sample reported higher compassion satisfaction, lower burnout and secondary traumatic stress, than those with less experience.

Moreover, with the intention of observing the relationship between burnout, self-esteem and compassion, Erkorkmaz et al. (2018) conducted an analytical cross-sectional study on nurses, finding that burnout was affecting both compassion satisfaction and personal accomplishment (subscale of the Maslach Burnout Inventory) (Maslach et al., 1997) negatively.

Using the Self-Compassion Scale (SCS)-Short Form (Raes et al., 2011) and the Barriers to Physician Compassion Questionnaire (Fernando and Consedine, 2014). Dev et al. (2018) evaluated the associations between burnout and barriers to compassion in a large sample of nurses. Their findings showed the expected association in which higher levels of burnout predicted greater barriers to compassion while higher levels of trait self-compassion were associated with lower burnout and predicted lower barriers to compassion.

#### About Mindfulness and Compassion Fatigue Correlation

Brown et al. (2017) explored the association between compassion fatigue and mindfulness in mental health professionals, considered that the purpose of the study was to explore whether there is an inverse relationship between the two variables, measured by the Five Facet Mindfulness Questionnaire (FFMQ) (Baer et al., 2008) and the ProQOL-5. The results showed a moderate, negative correlation between compassion fatigue and mindfulness. These findings suggest that high levels of compassion fatigue are associated to lower levels of mindfulness, meaning that mindfulness traits may be helpful in ameliorating compassion fatigue.

Similarly, Olson et al. (2014) collected data regarding burnout, emotional intelligence, empathy, mindfulness, self-compassion and resilience, in pediatric medicine residents of an urban children's hospital. Their measure was carried out also through the FFMQ and the SCS (Neff, 2003) and the results reported a negative association between mindfulness/self-compassion and burnout, with a positive association between the two and resilience factors and less emotional exhaustion. The authors suggest that both mindfulness and self-compassion may protect professionals' personal health and well-being.

Gracia-Gracia and Oliván-Blázquez (2017) analyzed the ability of self-compassion and mindfulness associated to burnout in nurses of intensive care units, also investigating the relationship between self-compassion as a positive mental state in association with mindfulness. The results of this study showed that compassion variables were predictive for burnout (emotional exhaustion, depersonalization and personal accomplishment), in particular the years of overall professional experience, self-kindness, self-judgement and the humanity-isolation factor of compassion. Furthermore, the mindfulness subscale appeared to have an inverse significant correlation with emotional exhaustion.

The research group of Montero-Marin et al. (2016), while confirming the validity of the burnout subtype model, assessed the explicative power of the self-compassion construct as a protective factor. According to a previous described study (Gracia-Gracia and Oliván-Blázquez, 2017), they used the SCS for this purpose. Their findings were that negative self-compassion dimensions might play an important role in the development of the burnout subtypes in Spanish HCPs, suggesting that negative self-compassion dimensions should be considered as vulnerability factors.

Silver et al. (2018) conducted a research with the aim of assessing relationships between mindfulness and some professional variables such as burnout, compassion fatigue, work engagement, and empathy on genetic counselors that provide immediate clinical aid to patients. They used the Mindfulness Attention Awareness Scale (MAAS) (Brown and Ryan, 2003) to evaluate mindfulness levels and the ProQOL-5 for burnout and compassion fatigue. Their findings showed that half of the participants reported engaging in activities which are typically associated with mindfulness (such as yoga, meditation, or breathing exercises); furthermore, there was a significant positive correlation between empathy/work engagement and mindfulness and moderate negative associations between mindfulness and burnout/compassion fatigue. According to some other studies, the authors suggest that mindfulness could affect positively professional satisfaction and quality of patient care.

### Effectiveness of MBSR, Mindfulness-Related, or Compassion-Related Interventions—Pre-post Design Studies

#### MBSR Intervention

Brady et al. (2012) assessed the impact of the MBSR program on managing work stress and improving patient outcomes in 16 hospital staff members consisting of psychiatrists, psychologists, nurses, social workers, mental health technicians, and activity therapists. The structured 4 week MBSR program was successful in influencing the levels of personal stress, mindfulness, and intrapersonal presence of inpatient psychiatric staff members.

Cohen-Katz et al. (2005) reported results exploring the effects of MBSR on 25 nurses. Treatment group subjects significantly decreased scores on 2 of 3 subscales of the Maslach Burnout Inventory compared to wait-list controls, while within-group comparisons for both groups pre-treatment and post-treatment revealed similar results. Changes were maintained as long as 3 months post-treatment.

Duarte and Pinto-Gouveia (2017) evaluated the effects of an MSBR program on 29 oncology nurses, with respect to 19 nurses of a waitlist comparison condition. Changes in mindfulness mediated changes in burnout, anxiety and stress, and satisfaction with life; changes in self-compassion mediated the influence of the intervention on burnout, depression, anxiety, stress and satisfaction with life; and psychological inflexibility mediated decreases in burnout, compassion fatigue, depression, and stress.

Lamothe et al. (2018) considered the feasibility and acceptability of an MBSR-based intervention and its impact on psychological variables, assessed pre- and post-intervention, like mindfulness, empathy, identification of one's own emotions and those of others, emotional acceptance and recognition of emotions in others, in two studies: in Study 1, 12 students completed the 8 week MBSR program, while in Study 2, 25 HCPs completed the 8 week MBSR program. Participants who completed the program improved on all measures except the identification of others' emotions and empathy.

Wylde et al. (2017) compared the effects of an 8 week MBSR intervention to a smartphone delivered mindfulness intervention, Headspace, an audio-guided mindfulness meditation program, in a group of 95 novice nurses, at the beginning of their internship and 3 months after entering the program, measuring compassion satisfaction, compassion fatigue, burnout, stress, trauma symptoms, and mindfulness variables. Nurses in the smartphone delivered mindfulness group showed significantly more “acting with awareness” and more “non-reactivity to inner experience” skills with respect to the MBSR group. The smartphone intervention group also reported more compassion satisfaction and less burnout. For novice nurses, smartphone delivered mindfulness interventions may be more beneficial.

A controlled mixed methods pilot study (Verweij et al., 2016) investigated the feasibility and effectiveness of MBSR on burnout, empathy, and work-related well-being, assessed before and after the intervention, in 43 general practitioner trainers of two Dutch hospitals. The MBSR group showed a greater reduction in depersonalization than the control group, while mindfulness and dedication increased significantly in the MBSR group than in the control group. There was no significant difference in empathy.

Raab et al. (2015) carried out a pilot study on the effects of an MBSR educational intervention on 22 female mental health professionals' self-compassion, perceived stress, burnout, and quality of life, assessed pre- and post-intervention. Changes in the SCS total score proved to be significant, while, regarding subscales, changes were significant in self-judgment, common humanity, isolation and overidentification.

#### Mindfulness-Related Interventions

Regarding mindfulness-related interventions, an evaluation of the impact of a group-based training program, known as “Occupational Mindfulness” (OM), on coping strategies and well-being of 34 employees within a disability service was carried out by Brooker et al. (2013). The program was positively evaluated by participants and found to be associated with significant increases in positive affect and the mindfulness facet of observing. Conversely, extrinsic job satisfaction showed a significant reduction from baseline to post-training, while negative affect, perceived stress, anxiety, and negative emotional symptoms showed a significant increase. Paradoxical increases of negative emotional symptoms are explained by considering that participants were developing higher levels of awareness of their current circumstances, whether positive or negative, internal or external. Mindfulness is not directly related to changing circumstances but about developing awareness to what those circumstances are.

A pre-post qualitative study investigated the effects of short-dose, 1–3 min-guided periods of mindfulness, conducted at the beginning of several staff meetings for 20 health professionals, which comprised social workers, therapists, nurses, doctors, administrative staff and leaders. The mindfulness facilitators were interviewed before the intervention and predicted that their groups would experience numerous intra- and interpersonal benefits. After implementation, they showed all of these benefits (Duggan and Julliard, 2018).

Farina et al. (2018) induced professional nurse educators to offer a 10–12 min mindfulness experiential learning session, during the institute's annual professional development forums, to 545 nurses. The nurse educator shared a biofeedback card and survey and asked the nurses to obtain and document their pre-practice and post-practice biofeedback card color and conclude the survey. The difference in the self-reported biofeedback card colors, indicating calm or relaxed states before and after the session, was significant.

Fernando et al. (2017) evaluated whether a brief mindfulness intervention increased compassionate responding to difficult patients and assessed whether the self-compassion trait moderated the impact of this experimental manipulation in a sample of 83 medical students. The intervention elicited mindfulness equivalently at both high and low levels of self-compassion. Furthermore, mindfulness predicted greater patient “liking” and “caring,” but only among students lower in self-compassion, while mindfulness predicted greater helping behavior, but primarily among students with higher self-compassion.

The Mindful Nursing Pilot Study (Horner et al., 2014) was a quasi-experimental research where 46 nurses belonging to a nursing unit participated in the 10 week mindfulness training program while an additional nursing unit served as the control group. Classes were held once a week and lasted 30 min. The group that underwent the intervention showed improvement in levels of mindfulness, burnout, and stress as well as patient satisfaction (though mindfulness and burnout scores did not reach significance) while the control group showed no significant differences.

Klatt et al. (2017) administered a brief experiential introduction to mindfulness to 286 HCPs, including dieticians, nurses, psychologists, and smoking cessation educators, measuring, during the meetings and at 3 month follow-up, participant's previous awareness and use of Complementary and Alternative Medicine techniques, and consequent probability of deepening the knowledge of these modalities for personal and professional use. Immediately after the experiential introduction 94.79% of respondents reported a probability to deepen the knowledge of mindfulness for personal use and 92.58% for professional use, while, at 3 month follow-up, 58% had used mindfulness personally and 28% reported that they had used mindfulness techniques professionally.

Pakenham (2015) investigated the effects of an Acceptance and Commitment Therapy (ACT) training, 12 2 h weekly workshops, on stress, therapist skills and characteristics, and the personal acquisition of ACT strategies in 32 clinical psychology trainees that completed measures of work-related stress, psychological distress, self-compassion, counseling self-efficacy, client-therapist alliance, acceptance and action, mindfulness, thought suppression and values, before and after university-based ACT training. Results showed that clinical psychology trainees reported improvements from before to after training on measures of counseling self-efficacy, client–therapist alliance, self-kindness, acceptance, defusion, mindfulness and values, and a marginally significant improvement on somatic symptoms, despite a trend toward increased work-related stress.

A pilot study (Pflugeisen et al., 2016), using a single-sample, pre-post study design, evaluated the feasibility of implementing an 8 week video-module based mindfulness pilot program aimed to reduce stress, ameliorate well-being, and develop mindfulness skills in 23 physicians in a community hospital setting. Participants experienced three 90 min in person trainings, weekly online video-module trainings, and weekly teleconference coaching calls. Physician stress, well-being (emotional exhaustion, depersonalization of patients, sense of personal accomplishment), and mindfulness skills (observing, describing, acting with awareness, acceptance without judgment) were evaluated. Significant reductions in stress, personal accomplishment, and emotional exhaustion and improvements in all mindfulness skills were observed at end-of-program.

Sansó et al. (2018), in a pre-post pre-experimental study, evaluated the outcome of a 6 week mindfulness training program on mindful attention, self-compassion, and professionals' quality of life in 36 HCPs of palliative care teams. After the intervention, there was an improvement of mindfulness, self-compassion, and burnout risk levels lowered, regardless of the profession.

Steinberg et al. (2016) explored the feasibility of a workplace intervention for improving resilience to stress of 32 of surgical intensive care unit HCPs, randomly assigned to an intervention or control group. The intervention consisted of a MBI including meditation, mild yoga movement, and music and was carried out in a group format 1 h a week for 8 weeks. Measures of burnout, compassion satisfaction, compassion fatigue and work engagement were obtained before and after the intervention. Work satisfaction improved significantly in the intervention group with no difference in the control group. Participants reported that recognizing their stress response was a main benefit of the intervention.

The study conducted by Taylor et al. (2016) examined the feasibility and impact of a 10 day mindfulness meditation intervention on 33 pediatric residents using a free smartphone application, Headspace. Measures of burnout and mindfulness were administered before and after the intervention. After the intervention, an increased percentage of residents perceived mindfulness as a useful intervention for patients and there was a significant increase in the number of residents who planned to discuss mindfulness as a therapeutic option for their patients, while there were no changes in burnout scores.

A field study (Wacker and Dziobek, 2018) evaluated a 3 day employee training in non-violent communication on non-violent communication skills, cognitive and emotional empathy, empathic distress and perceived social stressors at work in 29 HCPs. Participants filled out questionnaires before and 3 months after training. Results showed that communication skills were fostered in training participants, evidenced by the higher levels of emotion verbalization behavior and the enhanced use of non-violent communication at work. Empathic distress decreased, and the elevation of social stressors at work was hindered by enhanced emotion verbalization.

Wahl et al. (2018) aimed to conduct a peer support network pilot project through the inclusion of education/training, peer support and resiliency training and to investigate how interventions impact compassion satisfaction and compassion fatigue of 20 nurses, working in a community hospital, that completed pre-intervention and 6 week post-intervention surveys. Results revealed statistically significant improvements in compassion satisfaction and non-significant improvements in compassion fatigue.

Ofei-Dodoo et al. (2020) examined a group of 43 HCPs that participated in 8 week workplace, group mindfulness-based yoga intervention. Participants completed online measures regarding depression, anxiety, stress, resilience, burnout, and compassion at baseline and post-intervention. After the intervention, HCPs showed significant improvements on personal accomplishment, depression, anxiety, stress, perceived resilience, and compassion.

#### Compassion-Related Interventions

Thirteen nursing professionals underwent a stress reduction program, including mindfulness and loving kindness meditation. Quality of life assessment revealed significant increase as well as perceived stress, burnout, depression, and trait anxiety, while self-compassion did not show significant differences (Santos et al., 2016).

An observational, mixed-method pilot evaluation study (Orellana-Rios et al., 2017) enrolled 28 staff members of an interdisciplinary palliative care team and explored the feasibility and effectiveness of a 10 week group program with four aims: development of a mindful presence, cultivation of loving-kindness, Tong-len practice in difficult situations and the integration of these practices into daily work occupations. The investigated variables were burnout, perceived stress, anxiety, depression, somatization symptoms, emotion regulation, perceived job situation and goal attainment. Significant ameliorations were found in two of three burnout components (emotional exhaustion and personal accomplishment), anxiety, stress, two emotional regulation competences and joy at work. In addition, 85% of the individual goals were achieved.

A pilot study (Pfaff et al., 2017), with an embedded experimental mixed-methods design, evaluated the impact of 6 week formalized pilot compassion fatigue resiliency intervention on 32 HCPs participants at a regional cancer center. The impact of the intervention, in a pre- and post-intervention design, was evaluated on compassion fatigue and satisfaction, burnout, clinical stress and silencing responses. Participants showed diminished clinical stress at intervention completion, but no other overall changes.

### Effectiveness of MBSR, Mindfulness-Related or Compassion-Related Interventions—Cohort Design Studies

#### MBSR Intervention

Eliassen et al. (2016) examined the effect of two different training programs, namely two 8 week interventions of mindfulness training and affect-consciousness training, on mindfulness and on the perception of the ward atmosphere of two groups of HCPs, consisting of 27 and 23 participants, respectively. Measurements were taken at two baseline time points and four times following the intervention. Findings in this study indicate that MBSR staff training may give rise to beneficial changes in measures of Support, Program Clarity and Anger and Aggressive Behavior, as indicated by the Ward Atmosphere Scale (WAS) (Røssberg and Friis, 2003) subscales, suggesting that the MBSR intervention may modulate staffs' manner of relating to patients. Conversely, affect-consciousness staff training, as measured with the WAS, may strengthen Order and Organization, with an enduring effect up to 6 month follow-up. Differences on different subscales on the WAS may indicate that the affect-consciousness intervention is different in its effect as compared to the MBSR.

#### Mindfulness-Related Interventions

Research by Kemper's research group mainly investigated the impact of brief online mindfulness training on mindfulness and other psychological variables of HCPs. Kemper (2016) analyzed data from 178 HCPs and trainees who completed self-reflection exercises included in online mindfulness training, including three 1 h modules: introduction to mindfulness, mindfulness in daily life and mindful breathing and walking. Outcome data was related to mindfulness, measured with the Cognitive and Affective Mindfulness Scale–Revised (CAMS-R) (Feldman et al., 2006), the MAAS and the FFMQ. Concluding a brief, online training was associated with small but significant improvements in mindfulness scores, measured by all the three tools. In addition, there were no changes in improvement by gender, among the different HCPs. In a related prospective cohort study, Kemper and Rao (2016) investigated the effects of an online training program related to focused attention meditation, including three 1 h modules (introduction to stress, resilience and the relaxation response, clinical effects of the relaxation response and physiological effects of the relaxation response) on resilience, relaxation, stress, positive and negative affect and flourishing variables, assessed before and after training, of 379 HCPs. Even brief, online training was associated with small but significant improvements in relaxation, resilience, stress, positive and negative affect, and flourishing.

Luthar et al. (2017) reported on the effects of an intervention to foster resilience among women health care providers who are mothers at high risk for stress and burnout. 40 mothers were assigned randomly to either 12 weekly 1 h sessions of a structured, relational supportive intervention, the Authentic Connections Groups, including minimizing rumination, “good enough” mothering and shame (Carmassi et al., 2017a) vs. self-compassion, or to 12 weekly hours to be used as needed. Participants were evaluated at baseline, after the intervention, and 3 months follow-up on general symptomatology, depression, self-compassion and burnout plus plasma cortisol. After the intervention, results showed higher levels of improvements for mothers in the Authentic Connections Groups than control condition for depression and global symptoms. By 3 months follow-up, significant changes were found for depression and global symptoms and self-compassion, feeling loved, physical affection received, and parenting stress. Participants in the Authentic Connections Groups (but not control group) condition also showed significantly diminished cortisol levels at both after the intervention and follow-up.

O'Mahony et al. (2017) evaluated the preliminary outcomes of a group-based 9-session multimodal mindfulness training pilot designed to improve indicators of burnout and mental health symptomatology in 13 health care providers who interact with children in the context of end-of-life care. Measures were assessed before the program, at the program midpoint, and at the end of the program and were PTSD symptomatology, experiential avoidance, cognitive fusion, depression and burnout. Results revealed significant improvements in depressive and PTSD symptoms among HCPs.

Riley et al. (2016) carried out two studies to compare the impact of Cognitive Behavioral Stress Management (CBSM) and an 8 week Yoga-Based Stress Management (YBSM) interventions for HCPs. Study 1 evaluated YBSM intervention in 37 mental HCPs and gathered data regarding depression, anxiety, stress, health-related quality of life, coping skills, emotion regulation, mindfulness and self-compassion, pre- and post-intervention. Study 2 investigated YBSM and CBSM in 40 randomly assigned mental health care providers and collected data regarding depression, anxiety, stress, health-related quality of life, burnout, coping skills, and general health data at four time points. Results of Study 1 showed statistically significant increases in relaxation, coping confidence, self-kindness, self-compassion, general mental health, general physical health, and mindfulness, while results of Study 2 showed significant improvement in compassion satisfaction, depression, stress, heart rate, alcohol consumption, and burnout.

Suyi et al. (2017) examined the efficacy of a 6 week mindfulness program in improving mindfulness, compassion and self-compassion, and diminishing perceived stress and burnout, among 37 mental HCPs in Singapore. The program comprised 2 h sessions offered once a week and utilized a range of mindfulness techniques to instruct participants to cultivate compassionate and non-judgemental attitudes toward their inner experiences. Data was gathered at three time-points: pre- and post-intervention, and a 3 months follow-up. Participants showed significant amelioration in four of the five mindfulness domains (observe, describe, non-judge, non-react) and in compassion and self-compassion levels, and a significant decrease in stress, following mindfulness intervention. The improvements in mindfulness and self-compassion levels were maintained at three months follow-up. No significant difference was observed for burnout variables.

Wen et al. (2017) assessed how the self-guided, smartphone-based mindfulness app, Headspace, affects mindfulness, stress and negative emotions variables in 43 medical residents. Measures were collected before the intervention, at the midpoint (after 15 days), and at the end of the intervention (after 30 days). Results showed that both the mindfulness and positive affect scores showed a significant improvement with increasing use of the app, while the negative affect score did not show significant change.

#### Compassion-Related Interventions

Kemper et al. (2015) assessed the impact of online Mind-Body Skills (MBS) training on clinicians' and trainees' stress, mindfulness, and confidence in providing calm, compassionate care. MBS training (http://mind-bodyhealth.osu.edu) contained 12 1 h modules of continuing medical education–approved material organized in 4 general topics: focused attention meditation (relaxation response), mindfulness meditation, positive affect meditation (positive or sacred word, gratitude, and loving-kindness/compassion meditation) and guided imagery/hypnosis (autogenic training; guided imagery to prepare for surgery, procedures, or childbirth; and guided imagery for sleep and changing habits). Participants who engaged in MBS significantly improved in measures of stress, mindfulness, and confidence in offering calm, compassionate care. In a related prospective cohort study, Kemper and Khirallah (2015) assessed the effects of MBS training on mindfulness, resilience, and empathy of 513 HCPs. Completion of MBS training was related with significant ameliorations in stress, mindfulness, empathy and resilience, suggesting that this training is able to reach diverse HCPs.

Scarlet et al. (2017) studied the effects of an 8 week Compassion Cultivation Training (CCT) (Jazaieri et al., 2013) on work-related burnout, interpersonal conflict, mindfulness, self-compassion, fear of compassion and job satisfaction scores of 62 HCPs. The questionnaires were administered by email during the first, middle, and last weeks of CCT, as well as 1 month follow-up. Results showed significant improvements in participants' self-compassion, mindfulness, and interpersonal conflict levels.

## Discussion

This systematic review summarized the evidence regarding mindfulness- and compassion-related qualities of HCPs as well as potential effects of MBSR, mindfulness-related and compassion-related interventions on professionals' psychological variables like mindfulness, compassion, self-compassion and quality of life. The number of articles that met the inclusion criteria was 57 (4 RCTs, 23 studies with pre-post measurements, 12 cross-sectional studies, 11 cohort studies and 7 qualitative studies).

### Qualitative Studies

In regard of the qualitative studies, most of them investigated the relationship between mindfulness and compassion dispositions with burnout, stress levels, positive workplace relationships, and individualized patients care (Platt et al., 2015; Lyddy et al., 2016; McPherson et al., 2016; Hunter et al., 2018; Powell et al., 2018). From the examined studies, mindfulness dispositions appeared to be important in the fight against burnout and to manage effectively the stress response (Platt et al., 2015). Moreover, HCPs would seem to benefit more from the use of informal practices than from formal ones (Lyddy et al., 2016). As a result, the suggestion that we could gain from this information collection is to effectively increase mindfulness training in healthcare settings and provide professionals with less formal tools to tackle their job.

### Randomized Controlled Trials

With regard to the RCT studies, we highlight that MBSR or mixed mindfulness interventions resulted to be more effective than controls in samples of doctors and HCPs in three of the four examined researches. In particular, mindfulness interventions were most effective on stress (Amutio et al., 2015; Ireland et al., 2017), job burnout (Amutio et al., 2015; Ireland et al., 2017), and safety compliance at work (Valley and Stallones, 2017). Amutio et al. (2015) also highlighted the importance of a maintenance programme after the mindfulness intervention.

### Cross-Sectional Design Studies

With reference to the cross-sectional selected studies, we observed various researches linking compassion fatigue/compassion satisfaction (risk and prevalence) with violence exposure, coping mechanisms, burnout and bereavement overload (Brown et al., 2017; Allie et al., 2018; Copeland and Henry, 2018). With respect to this topic, the results obtained are somewhat controversial; on the one hand, we observed a confirmation of a high presence of burnout, compassion fatigue and secondary traumatic stress in the investigated samples. On the other hand, many studies have observed internal variations concerning these variables, depending on gender or years of experience at work (Al-Majid et al., 2018; Roney and Acri, 2018; Wijdenes et al., 2019). Supposing that compassion fatigue is a consequence of burnout, some studies have also pointed out the positive correlation between burnout and compassion barriers or between bereavement overload and emotional detachment (Gracia-Gracia and Oliván-Blázquez, 2017; Dev et al., 2018). These findings should elicit some reflections on the role played by the excessive stress load on the behavior of health personnel, redesigning both the therapeutic and interventional approach to this topic.

Moreover, as far as correlation between mindfulness and compassion is concerned, findings show that mindfulness approach may be useful in mitigating the negative aspect of compassion fatigue, which is, in turn, predictive for burnout (Brown et al., 2017). Consequently, both mindfulness and compassion satisfaction represent a protective factor on the HCPs' well-being and quality of patient care. Mindfulness also demonstrated a positive correlation with resilience, empathy, work engagement, and less emotional exhaustion (Olson et al., 2014; Silver et al., 2018). Finally, these findings should elicit a reflection about the negative impact of self-compassion dimensions, which could be studied as a vulnerability factor.

### MBSR and Mindfulness-Related Interventions

Regarding pre-post design research, 7 studies investigated the effects of MBSR intervention on HCPs. MBSR proved to be effective in maintaining (Cohen-Katz et al., 2005) or improving mindfulness levels (Brady et al., 2012; Verweij et al., 2016; Duarte and Pinto-Gouveia, 2017; Lamothe et al., 2018), and an MBSR educational intervention was effective at improving self-compassion levels (Raab et al., 2015). In novice nurses, a smartphone-delivered audio-guided mindfulness meditation program, Headspace, resulted in more acting with awareness compared to the MBSR group (Wylde et al., 2017). It may be hypothesized that for novice meditators, that are approaching mindfulness, a progressive approach to meditation may be helpful. In a cohort study, MBSR seems to promote beneficial changes in measures of support, program clarity, anger and aggressive behavior suggesting that the MBSR intervention may be helpful at regulating staffs' relational abilities with patients (Eliassen et al., 2016).

Studies employing intervention protocols that were different from standard MBSR, were carried out with very different mindfulness-related interventions. Interventions that were found to be effective at improving mindfulness levels were 1–3 min-guided periods of mindfulness (Duggan and Julliard, 2018), 10 min mindfulness induction exercises (Fernando et al., 2017), a 10 day mindfulness meditation intervention using a free smartphone application (Headspace) (Taylor et al., 2016), 6 week mindfulness training (Sansó et al., 2018), an 8 week video-module based mindfulness program (Pflugeisen et al., 2016) and an ACT training (Pakenham, 2015), while a 10 week mindfulness training program, the Mindful Nursing Pilot Study, resulted in mindfulness improvements that were not statistically significant (Horner et al., 2014), possibly because of sessions that lasted 30 min, instead of 60 or 90 min. OM was found to increase positive affect and the mindfulness facet of observing together with a paradoxical increase of negative emotional symptoms, possibly due to a greater awareness of participants current circumstances (Brooker et al., 2013). An 8 week MBI was found to be effective on work satisfaction but not on compassion satisfaction or compassion fatigue (Steinberg et al., 2016), while a peer support network pilot project resulted in statistically significant improvements in compassion satisfaction and non-significant improvements in compassion fatigue (Wahl et al., 2018). An 8 week supervised workplace, group mindfulness-based yoga intervention was able to improve compassion levels (Ofei-Dodoo et al., 2020), while A 3 day employee training in non-violent communication was able to reduce empathic distress (Wacker and Dziobek, 2018) and a brief experiential introduction to mindfulness increased the likeliness to investigate, or to use, mindfulness for personal or for professional use (Klatt et al., 2017). Ten to twelve minutes of mindfulness experiential learning session was found to produce a statistically significant difference in the self-reported biofeedback card colors indicating calm or relaxed states before and after the session (Farina et al., 2018). Analogously, cohort studies showed that mindfulness-related interventions were found to be effective at improving depression, global symptoms, self-compassion, feeling loved, physical affection received, parenting stress, and cortisol levels (Luthar et al., 2017), at reducing depressive and PTSD symptomatology (O'Mahony et al., 2017), at improving compassion satisfaction, depression, stress, heart rate, alcohol consumption and burnout (Riley et al., 2016) and at increasing four of the five mindfulness domains (observe, describe, non-judge, non-react) and compassion and self-compassion levels (Suyi et al., 2017). Kemper's research group investigated the effects of a brief online mindfulness training that was able to improve mindfulness scores, measured by CAMS-R, MAAS, and FFMQ (Kemper, 2016), and relaxation, resilience, stress, positive and negative affect, and flourishing (Kemper and Rao, 2016), while the smartphone-based mindfulness app, Headspace, was able to increase mindfulness and positive affect scores (Wen et al., 2017).

### Compassion-Related Interventions

Compassion-related interventions, that mainly included loving-kindness meditation (Santos et al., 2016; Orellana-Rios et al., 2017) or a formalized pilot compassion fatigue resiliency intervention (Pfaff et al., 2017), were found to be effective at improving quality of life (Santos et al., 2016), burnout components of emotional exhaustion and personal accomplishment (Orellana-Rios et al., 2017) and at reducing clinical stress (Pfaff et al., 2017). No other main significant changes were observed. In cohort studies, Kemper's research group found that online MBS training was effective at significantly improving stress, mindfulness, and confidence in providing calm, compassionate care (Kemper et al., 2015) and at providing significant ameliorations in stress, mindfulness, empathy and resilience (Kemper and Khirallah, 2015). CCT (Jazaieri et al., 2013) was found to significantly enhance participants' self-compassion, mindfulness, and interpersonal conflict scores (Scarlet et al., 2017).

### MBSR, Mindfulness,- and Compassion-Related Interventions

Taken together these results suggest that MBSR intervention is able to improve, and maintain, mindfulness levels, self-compassion levels and to improve burnout, depression, anxiety, stress (Lamothe et al., 2016). MBSR intervention has been shown to be able to modulate anger and aggressive behavior as well, with a suggestion that a smartphone-delivered audio-guided mindfulness meditation program, Headspace, may be helpful for novice HCPs.

The most frequently employed interventional strategies were mindfulness-related trainings (Fox et al., 2018) that proved to be effective at improving nurses' mental health significantly (Guillaumie et al., 2017), for treating burnout in occupational therapy professionals (Luken and Sammons, 2016) and at reducing stress among HCPs (Burton et al., 2017). Overall, as also outlined by West et al. (2016), mindfulness-related interventions, even of brief duration (Gilmartin et al., 2017), were effective at improving mindfulness levels, self-compassion, depression, global symptoms, feeling loved, physical affection received, parenting stress, cortisol levels, work and compassion satisfaction and at increasing the likeliness to investigate, or to use, mindfulness for personal or for professional use, but not at improving compassion fatigue (Steinberg et al., 2016; Wahl et al., 2018). Though the true prevalence of burnout (Rotenstein et al., 2018), compassion fatigue, secondary traumatic stress and vicarious trauma in HCPs (Mol et al., 2015) remains open for discussion, compassionate care is fundamental for better clinical and patient outcomes, but during healthcare provision it can be hampered by several factors. It has been highlighted that stress and negative affect, on which MBSR is particularly effective (Lamothe et al., 2016), were moderately positively associated with compassion fatigue, and that positive affect also had a moderately positive relationship with compassion satisfaction (Zhang et al., 2018). As for the neurobiological studies (Hölzel et al., 2013; Taren et al., 2013, 2015; Engen and Singer, 2015), mindfulness seems to be effective at improving negative affect and, possibly, compassion fatigue, while compassion satisfaction may be related to cultivation of positive affect. In this regard, CCT (Jazaieri et al., 2013; Scarlet et al., 2017) and MBIs (Wasson et al., 2020) have been shown to improve participants' self-compassion, mindfulness, and interpersonal conflict scores.

Remarkably, Egan et al. (2019) reported that HCPs are aware of the burnout potential and their experiences of the necessity to access help to cope with a demanding environment. However, HCPs were not tired of being compassionate (compassion fatigue), but rather, tired of having to overcome the organizational barriers to being compassionate. Participants did not state that they were tired of caring, but they were tired of not being able to care as they would like to. In this regard, MBSR, mindfulness- and compassion-related interventions may prevent HCPs' burnout and promote their enduring compassion and caring.

## Limitations and Future Research

The search strategy used in this review limited the search with the inclusion criteria of English or Italian language and peer-reviewed articles only. Consequently, though an extensive search was carried out, it is not correct to claim that the review is exhaustive given the papers excluded that were written in other languages or articles published in other formats (e.g., unpublished theses).

Despite these limitations, this systematic review presents a synthesis of mindfulness and compassion characteristics of the healthcare professionals and of MBSR, MBIs, and CBIs that have been delivered specifically to this professional population. Findings of the review depict the potential of MBSR, MBIs and CBIs as a tool for enhancing professionals' mindfulness, self-compassion and quality of life. Considering the differences of methodological approach and intervention protocols of the existing MBSR, MBIs, and CBIs studies, for future research it may be suggested to carry out studies that combine structured mindfulness and compassion cultivation trainings (e.g., mindful self-compassion program; Neff and Germer, 2013) to enhance the effects of interventions, to limit the variability of intervention protocols and to improve data comparability.

## Data Availability Statement

All datasets generated for this study are included in the article/supplementary material.

## Author Contributions

CC designed and executed the study, assisted with the data analyses, and wrote the paper. RC collaborated with the design and writing of the study. GO analyzed the data and wrote part of the results. AG and MD collaborated in the writing and editing of the final manuscript. AP collaborated with the design and writing of the study. All authors approved the final version of the manuscript for submission.

## Conflict of Interest

The authors declare that the research was conducted in the absence of any commercial or financial relationships that could be construed as a potential conflict of interest.
